# Phase-Field Simulation of Precipitation and Grain Boundary Segregation in Fe-Cr-Al Alloys under Irradiation

**DOI:** 10.3390/nano14141198

**Published:** 2024-07-14

**Authors:** Xuxi Liu, Wenlong Shen, Wenbo Liu

**Affiliations:** 1 Department of Nuclear Science and Technology, Xi’an Jiaotong University, Xi’an 710049, China; crescent46883386@stu.xjtu.edu.cn (X.L.); s2054283224@stu.xjtu.edu.cn (W.S.); 2 Shaanxi Key Laboratory of Advanced Nuclear Energy and Technology, Xi’an Jiaotong University, Xi’an 710049, China

**Keywords:** phase-field simulation, Fe-Cr-Al alloy, grain boundary, precipitation, segregation

## Abstract

A phase-field model for the precipitation of Fe-Cr-Al alloy is established incorporating grain boundary (GB) effects and irradiation-accelerated diffusion. The radiation source and grain boundary effect are incorporated to broaden the applicability of the Fe-Cr-Al precipitated phase-field model. The model is firstly employed to simulate the precipitation of the Cr-rich α’ phase in a single-crystal alloy. The precipitation rate and the size distribution of the precipitated phase were analyzed. Subsequently, the model is utilized to simulate segregation at GBs in a double-crystal system, analyzing the enrichment of Cr and depletion of Al near these boundaries. The simulation results are consistent with experimental observations reported in the references. Finally, the model is applied to simulate the precipitation in a polycrystalline Fe-Cr-Al system. The simulated results revealed that the presence of GBs induces the formation of localized regions with enhanced Cr and Al content as well as depleted zones adjacent to these boundaries. GBs also diminish both the quantity and precipitation rate of the formed phase within the grains.

## 1. Introduction

Fe-Cr-Al alloy exhibits excellent corrosion resistance, radiation resistance, and mechanical strength [1,2,3], making it a promising candidate as a structural material for the next generation of nuclear reactors, such as sodium fast reactors and lead-cooled fast reactors [4,5]. Due to its exceptional performance in high-temperature steam environments, Fe-Cr-Al alloys are also being considered for accident-resistant fuel cladding applications [6]. However, the mechanical properties of Fe-Cr-Al alloys have been observed to undergo embrittlement and hardening upon re-irradiation [7]. Increasing attention has been devoted to the investigation of intragranular precipitation and segregation at the grain boundary (GB) of the α’ phase (Cr concentration up to 45 at.%), which ultimately leads to a deterioration in the mechanical properties of α-BCC Fe-Cr-Al alloy [8]. Ejenstam et al. [9] found that the Fe-21 wt.% Cr-5 wt.% Al alloy is readily embrittled at 773 K. Due to the lattice structure of the precipitated phase which is different from the matrix and its very fine microstructure (2~5 nm), it is easy to produce stress concentration at the precipitated phase, resulting in crack nucleation and plastic fracture [8]. The influence of irradiation-induced vacancies and dislocations is of paramount importance [10]. It is necessary to further study the behavior of defects in Fe-Cr-Al under irradiation.

The extensively investigated microstructures of α−α’ phase separation induced by irradiation have been the focus of recent research [11,12]. The experimental results demonstrate that irradiation conditions significantly enhance the precipitation of the Cr-rich α’ phase in Fe-Cr-Al alloy and its segregation along the GBs [2,13]. For instance, Capdevila et al. [14,15] conducted a study on the miscibility gap of the PM 2000™ ODS alloy (Fe-20 at.% Cr-10 at.% Al). It was observed that compared to the Fe-20 at.% Cr alloy, the critical temperature decreased while the miscibility gap narrowed. In Priyam’s work [16], the segregation of the α’ phase in candidate Fe-Cr-Al alloy for advanced fuel cladding was systematically investigated under irradiation conditions. The alloys studied included C06M, C35M, C36M, and C37M with chromium content ranging from 9 to 12 wt.% and aluminum content ranging from 5 to 7 wt.%. In Miaosen’s work [17], the precipitates of Fe-Cr-Al alloy are explored using the molecular dynamics method and ab initio energy calculation. The role of Al in the segregation of Fe-Cr-Al alloy is discussed in their result. However, the current study still lacks a computational model capable of addressing the long-term irradiation-induced precipitation and segregation of Fe-Cr-Al, integrating both irradiation and GB as key factors.

In the past few decades, the phase-field method has already successfully been employed to simulate the precipitate in stainless steel for reactors including Fe-Cr-Al alloys [18,19,20]. Yang et al. [21,22] investigated the effects of GB and irradiation factors on the precipitation and segregation of Fe-Cr binary alloy. Their findings demonstrated the significance of integrating the GB effect with the precipitation of the second phase in Fe-Cr-Al. Chen et al. [23] investigated the precipitation of Cr-rich α’ precipitates in Fe-35Cr-10Al at various temperatures and conducted a statistical analysis on the distribution characteristics of precipitates across different temperature conditions. Lee et al. [24] investigated the precipitation of the Cr-rich α’ phase in Fe-Cr-Al alloys with varying Al contents and conducted a statistical analysis on the distribution characteristics of the precipitated phase. However, the existing phase-field models still lack the function to consider the two crucial factors of irradiation and GB into Fe-Cr-Al alloy. Therefore, it is imperative to develop a comprehensive phase-field model that encompasses precipitation and segregation phenomena in Fe-Cr-Al alloy under irradiation and at GBs.

In this work, a phase-field model for the precipitation and GB segregation in Fe-Cr-Al alloy is established incorporating irradiation-accelerated diffusion and GB effects. Simulations are conducted to investigate the behavior of the Cr-rich α’ phase under varying levels of irradiation intensity in a single-crystal structure, enabling calculation of the temporal distribution of precipitated phases. The influence of irradiation conditions on the precipitation of the α’ phase is analyzed based on the simulation results. Subsequently, simulations are performed to explore the segregation of Fe-10Cr-6Al alloy in double- and polycrystal systems. The impact of GBs on the precipitation of the α’ phase is analyzed based on the simulation results as well. The simulation results exhibit excellent concordance with the experimental findings.

## 2. Method

### 2.1. Phase-Field Parameters

**Figure 1:**
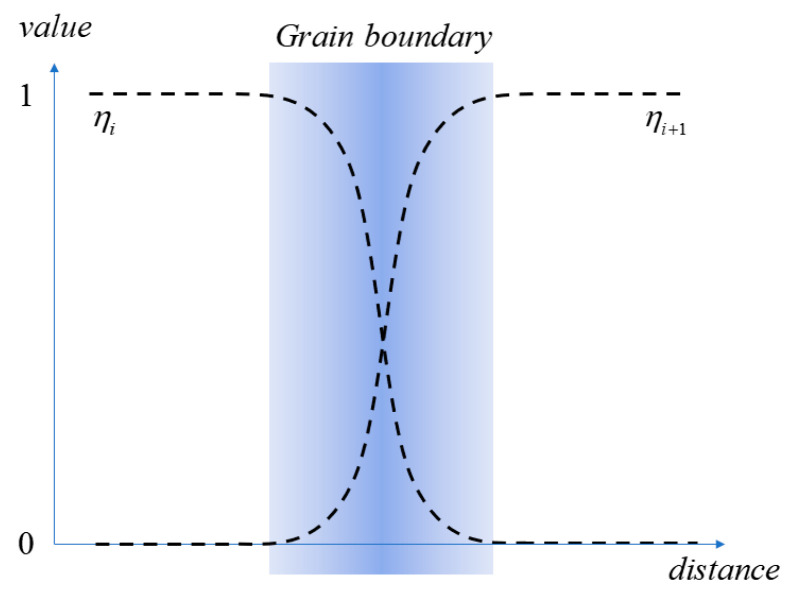
Phase-field parameters and the initial distribution at GBs (the dotted black lines represent the grain order parameters, while the blue gradient indicates grain boundaries).

The schematic diagram in Figure 1 illustrates the phase-field variables and their corresponding values, which are introduced to construct a phase-field model. Specifically, phase-field variables, namely, *η_i_* and *c_j_* are employed for this purpose. The variation in the orientation field is represented by the orientational order parameters *η_i_*, enabling differentiation between grains with different orientations in Fe-Cr-Al alloys where *i* represents the *i*-th grain. In grain *i*, *η_i_* has a value of 1, whereas in other grains, it takes on a value of 0. At the GB, *η_i_* exists in continuous variation from 0 to 1. The concentration field variable *c_j_* signifies the fraction of the element within the entire simulation system including Fe, Cr, Al, and vacancy. The value of *c_j_* is determined by element relative concentration and is initially set as uniformly distributed.

### 2.2. Total Free Energy Function

The total free energy F of Fe-Cr-Al alloys under the irradiation environment is expressed as Equation (1) including the chemical free energy *f_chem_*, GB energy *f_gb_*, and interfacial energy *f_grad_* [25,26].

(1) $$F=\int_{V}\frac{1}{V_{m}}\left[f_{chem}+f_{gb}+f_{grad}\right]dV$$

where the interfacial energy is a function of the spatial gradient of each phase-field variable as follows:

(2) $$f_{grad}=\kappa_{Cr}{\left(\nabla c_{Cr}\right)}^{2}+\kappa_{Al}{\left(\nabla c_{Al}\right)}^{2}+\kappa_{v}{\left(\nabla c_{v}\right)}^{2}+\sum_{i}\kappa_{\eta }{\left(\nabla c_{\eta }\right)}^{2}$$

where *κ_Cr_*, *κ_Al_*, *κ_v_*, and *κ_η_* are the gradient energy coefficients of Cr, Al, vacancy, and GB. *κ_Cr_* = *κ_Al_* = $\frac{1}{6}a_{0}^{2}L_{FeCr}$ [23,27], *κ_v_* = 6.91 × 10^−10^ J·m^−1^ [28], and *κ_η_* = 2 × 10^−10^ J·m^−1^ [22]. $a_{0}=c_{Fe}^{0}a_{Fe}+c_{Cr}^{0}a_{Cr}+c_{Al}^{0}a_{Al}$, $c_{Fe}^{0}$, $c_{Cr}^{0}$, and $c_{Al}^{0}$ are the initial relative concentrations of Fe, Cr, and Al, and $a_{Fe}$, $a_{Cr}$, and $a_{Al}$ are the lattice parameters of the pure elements. *a_Fe_* = 2.866 × 10^−10^ m. *a_Cr_* = 2.882 × 10^−10^ m and *a_Al_* = 4.050 × 10^−10^ m.

GB energy *f_gb_* is introduced as follows:

(3) $$f_{gb}=m(\phi )\left[\frac{1}{4}+\sum_{i}\left(\frac{\eta_{i}^{4}}{4}-\frac{\eta_{i}^{2}}{2}\right)+W\sum_{i}\sum_{j}\eta_{i}^{2}\eta_{j}^{2}\right]$$

where *W* represents the energy barrier at the GB. *W* = 4.0 × 10^9^ J·m^−3^ [22]. *m*(*ϕ*) is the function that couples the concentration and the orientational order parameters and is written as [29]:

(4) $$m(\phi )=1+0.1\phi^{2}-6\phi^{2}{\left(1-\phi \right)}^{2}$$

This form of *m*(*ϕ*) is introduced to explain the energy change at the grain boundary due to the concentration field changing from the *α* phase to the *α’* phase. The variable *ϕ* related to the concentration evaluates the process of transition from the *α* phase to the *α’* phase. The variable *ϕ* related to the concentration *m*(*ϕ*) takes the minimum at near *ϕ =* 0.5 to achieve less energy loss at the grain boundaries [30].

In this work, *ϕ* is established based on the weighted average of the initial concentrations of Al and Cr, depending on where its concentration lies between the *α* phase and *α’* phase as follows:

(5) $$\phi =\frac{c_{Cr}^{0}}{c_{Cr}^{0}+c_{Al}^{0}}\frac{c_{Cr}-c_{Cr}^{e}}{c_{Cr}^{e2}-c_{Cr}^{e}}+\frac{c_{Al}^{0}}{c_{Cr}^{0}+c_{Al}^{0}}\frac{c_{Al}-c_{Al}^{e}}{c_{Al}^{e2}-c_{Al}^{e}}$$

where $c_{Cr}^{0}$ and $c_{Al}^{0}$ are the initial alloy relative concentrations of Cr and Al. $c_{Cr}^{e}$ and $c_{Cr}^{e2}$ are the equilibrium concentrations of Cr in phase *α* and phase *α’*. $c_{Al}^{e}$ and $c_{Al}^{e2}$ are the equilibrium concentrations of Al in phase *α* and phase *α’* as well.

The chemical free energy *f_chem_* is introduced using molar Gibbs energy and is written as follows:

(6) $$\begin{array}{cc} f_{chem} & =c_{Fe}G_{Fe}^{0}+c_{Cr}G_{Cr}^{0}+c_{Al}G_{Al}^{0}+c_{v}E_{v}^{f}\\ & +\;L_{FeCr}c_{Fe}c_{Cr}+L_{FeAl}c_{Fe}c_{Al}+L_{CrAl}c_{Cr}c_{Al}\\ & +\;RT(c_{Fe}\ln c_{Fe}+c_{Cr}\ln c_{Cr}+c_{Al}\ln c_{Al}+c_{v}\ln c_{v}) \end{array}$$

where $c_{Fe}$ is the concentration of Fe and is conserved as $c_{Fe}=1-c_{Cr}-c_{Al}-c_{v}$ *E_v_^f^* = 1.12 eV is the formation energy of vacancy [28]. $G_{Fe}^{0}$, $G_{Cr}^{0}$, and $G_{Al}^{0}$ are the molar Gibbs free energies of pure Fe, Cr, and Al. *L_ij_* is the interaction parameter between elements *i* and *j*. *R* = 8.314 J·mol^−1^·K^−1^ is the universal gas constant. Temperature *T* is chosen as 773 K [9] in this work. The values used in *f_chem_* are listed in Table 1.

### 2.3. Evolution Governing Equation

In this work, a phase-field governing equation with a diffusion pair is introduced to describe the microstructure evolution of the Cr-enriched α′ phase in Fe-Cr-Al alloys. In the phase-field equation, the energy-driven diffusion of conserved concentration field variables is represented by the Cahn–Hilliard equation.

Due to the low concentration, the vacancy itself does not actively participate in the diffusion couple. Therefore, the diffusion of vacancies is still governed by the classical form of the Cahn–Hilliard equation as follows [28]:

(7a) $$\frac{\partial c_{v}}{\partial t}=\nabla M_{v}\nabla \frac{\delta F}{\delta c_{v}}+P_{v}-S_{v}$$

where *M_v_* is mobility of vacancy. $P_{v}=\sigma \cdot \phi_{dpa}$ is the production rate of vacancy, where $\phi_{dpa}$ is the irradiation dose rate and the value of $\phi_{dpa}$ is changed in subsequent simulations as the object of parameter sensitivity analysis. *σ* is the number of irradiated vacancies displaced per irradiation atom (dpa) [28]. *S_v_* = 3.01 × 10^−13^ m^−2^ is the sink strength of vacancy [33].

In the diffusion pair model, the Cahn–Hilliard equation is extended to the following form [34,35].

(7b) $$\frac{\partial c_{Cr}}{\partial t}=\nabla \left(M_{Cr,Cr}\nabla \frac{\delta F}{\delta c_{Cr}}+M_{Cr,Al}\nabla \frac{\delta F}{\delta c_{Al}}\right)$$

(7c) $$\frac{\partial c_{Al}}{\partial t}=\nabla \left(M_{Al,Al}\nabla \frac{\delta F}{\delta c_{Al}}+M_{Al,Cr}\nabla \frac{\delta F}{\delta c_{Cr}}\right)$$

where *M_ij_* is the Onsager coefficient determained by the atomic mobility as follows [36]:

(8a) $$M_{Cr,Cr}=M_{Fe}c_{Fe}c_{Cr}c_{Cr}+M_{Cr}{\left(1-c_{Cr}\right)}^{2}c_{Cr}+M_{Al}c_{Al}c_{Cr}c_{Cr}$$

(8b) $$M_{Al,Al}=M_{Fe}c_{Fe}c_{Al}c_{Al}+M_{Cr}{\left(1-c_{Al}\right)}^{2}c_{Al}+M_{Al}c_{Al}c_{Al}c_{Cr}$$

(8c) $$M_{Al,Cr}=M_{Cr,Al}=M_{Fe}c_{Fe}c_{Cr}c_{Al}-M_{Cr}(1-c_{Cr})c_{Cr}c_{Al}+M_{Cr}(1-c_{Al})c_{Cr}c_{Al}$$

where *M_i_* = *D_i_V_m_*/*RT* represents the atomic mobility of element *i*, *D_i_* is the diffusivity of element *i*, and *i* =Fe, Cr, Al, and *v* for vacancy. *V_m_* = 7.0 × 10^−6^ m^3^/mol is the atomic volume.

The diffusion coefficients used in this work’s simulation calculations are shown in Table 2.

The vacancy generated by irradiation will significantly enhance the mobility of alloy elements. The irradiation-enhanced diffusion coefficient can be rewritten as $M_{i}=M_{i}^{0}\cdot c_{v}/c_{v}^{e}$, where $c_{v}^{e}$ is the thermodynamic equilibrium concentration of vacancy. The vacancy concentration $c_{v}$ under irradiation conditions is expected to surpass that of $c_{v}^{e}$, leading to enhanced mobility of the element concentration and consequently accelerating the rate of precipitation. In this work, $c_{v}^{e}$ is chosen from the reference experiment observation results as $c_{v}^{e}$ = 1.62 × 10^−5^.

The time evolution of orientational order parameters *η_i_* are gravened by the Allen–Cahn equation as follows [39]:

(9) $$\frac{\partial \eta_{i}}{\partial t}=-L\nabla \frac{\delta F}{\delta \eta_{i}}$$

where *L* is there relaxation coefficient for the order parameter.

### 2.4. Numerical Method and Parameters

The variables and constants in the phase-field equations need to undergo dimensionless processing in order to facilitate numerical simulation on computers. The dimensionless processing in this work encompasses the equations $t^{*}=tD_{0}/{\left(\Delta l\right)}^{2}$, $\nabla^{*}=\partial /\partial l^{*}$, $r^{*}=r/\Delta l$, $M^{*}=MRT/D_{0}$, $\kappa^{*}=\kappa /RT{\left(\Delta l\right)}^{2}$, $f_{bulk}^{*}=f_{bulk}/RT$, and $f_{grad}^{*}=f_{grad}/RT$, where $D_{0}$ = 1 × 10^−25^ m^2^/s is a characteristic diffusion coefficient used to nondimensionalize the governing equations. Δ*l* is the grid scale and is valued as the average lattice constant $a_{0}=c_{Fe}^{0}a_{Fe}+c_{Cr}^{0}a_{Cr}+c_{Cr}^{0}a_{Al}$. In this work, the calculated time step is chosen as 0.001t^*^ which corresponds to the physical time of ~11.2 s. Because the precipitate of Fe-Cr-Al alloy has a slow time characteristic, the simulated step size of this work will exceed up to 10^6^ simulation steps.

In this work, a semi-implicit Fourier spectral technique is employed to improve the numerical efficiency. In the Fourier space, the physical quantities in the time domain are transformed into their corresponding counterparts in the frequency domain, and the evolution equation is computed within Fourier space. In Fourier space, after dimensionless processing, Equation (7a–c) is rewritten in the following form.

(10a) $$\frac{\partial {\tilde{c}}_{v}}{\partial t^{*}}=ik{\left[M_{v}^{*}ik^{\prime }{\left(\frac{\delta F^{*}}{\delta c_{v}}\right)}_{k^{\prime }}\right]}_{k}+P_{v}-S_{v}$$

(10b) $$\frac{\partial {\tilde{c}}_{Cr}}{\partial t^{*}}=ik{\left[M_{Cr,Cr}^{*}ik^{\prime }{\left(\frac{\delta F^{*}}{\delta c_{Cr}}\right)}_{k^{\prime }}+M_{Cr,Al}^{*}ik^{\prime }{\left(\frac{\delta F^{*}}{\delta c_{Al}}\right)}_{k^{\prime }}\right]}_{k}$$

(10c) $$\frac{\partial {\tilde{c}}_{Al}}{\partial t^{*}}=ik{\left[M_{Al,Al}^{*}ik^{\prime }{\left(\frac{\delta F^{*}}{\delta c_{Al}}\right)}_{k^{\prime }}+M_{Al,Cr}^{*}ik^{\prime }{\left(\frac{\delta F^{*}}{\delta c_{Al}}\right)}_{k^{\prime }}\right]}_{k}$$

where *i* is the imaginary unit and *k* = (*k*_1_, *k*_2_) is the vector in the Fourier space. ${\tilde{c}}_{i}$ and ${\left(K\right)}_{k}$ are the Fourier transforms from real space to the Fourier space of *c_i_* and expression *K*. ${\left(K\right)}_{k^{\prime }}$ is the inverse Fourier transform from the Fourier space to real space for expression *K*. In this work, the severe time-step constraint [40] $\Delta t\kappa k^{4}\leq 1$ is satisfied. The resulting evolution outcomes ${\tilde{c}}_{i}$ in the frequency domain are inversely transformed back to their original representation *c_i_* in the time domain, thereby significantly enhancing computational efficiency.

## 3. Result and Discussion

### 3.1. Irradiation-Accelerated Evolution of the α′ Phase

**Figure 2:**
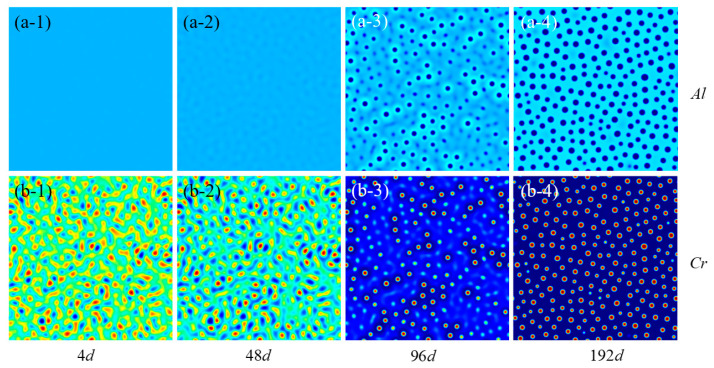
Plots of the Cr and Al concentrations in the evolution of Fe-Cr-Al decomposition at different times (**a-1**–**a-4**) represent the concentration distribution of Al, while (**b-1**–**b-4**) depict the concentration distribution of Cr at different time. Note that the blue region indicates a lower element relative concentration, while the red areas represent a higher element relative concentration.

**Figure 3:**
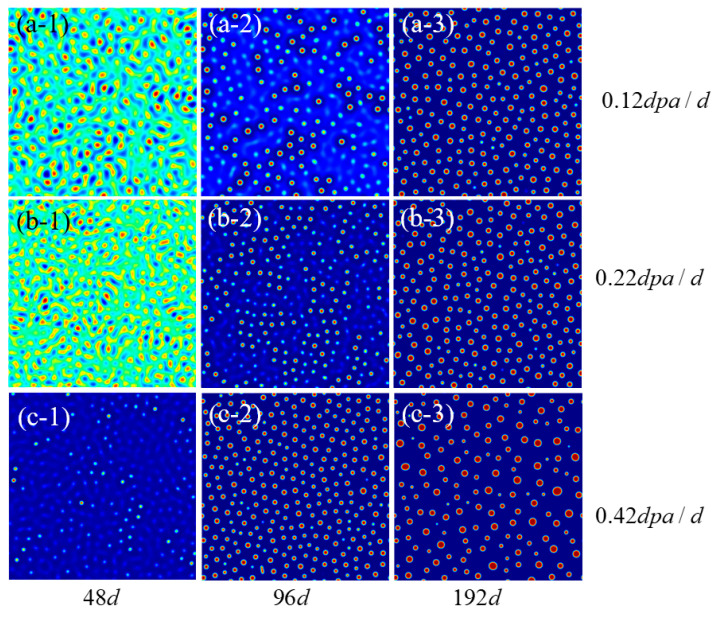
The Cr concentration distribution results at different irradiation intensities after different times (**a-1**–**a-3**,**b-1**–**b-3**,**c-1**–**c-3**) represent the concentration distribution of Cr under the irradiation intensities of 0.12 dpa/d, 0.22 dap/d, and 0.42 dpa/d, respectively. Note that the blue region indicates a lower element relative concentration, while the red areas represent a higher element relative concentration.

The model was first applied to investigate the precipitate of Fe-Cr-Al alloy in a single crystal. The evolution of Cr and Al of Fe-15Cr-6Al in a single crystal under an irradiation of 0.12 dpa/d are illustrated in Figure 2. The simulation size is set as 512 × 512 discrete grids, corresponding to an actual size of 306.2 × 306.2 nm. The simulation time scale is chosen as 2 × 10^6^ calculation steps, representing 259.26 days of actual physical time. The blue region indicates a lower element relative concentration, while the red areas represent a higher element relative concentration. Based on the simulation results, the alloy undergoes precipitation to form a spherical Cr-rich α’ precipitated phase through precipitation. Within this *α’* phase, the concentration of Cr is elevated and that of Al is reduced compared to the matrix phase. A time region for concentrated precipitation exists (Figure 2(a-3,b-3)) [13]. Prior to reaching this time region, the formation of the α’ phase is absent despite the aggregation of Cr elements. However, once this time region is reached, the rapid formation of the α’ phase occurs and it predominantly occupies the Cr element in the alloy. Subsequently, beyond this time region, there is only gradual growth instead of the further production of Cr-rich α’ precipitates [7].

The precipitate of Fe-15Cr-6Al is analyzed after subjecting it to different irradiation intensities for the same aging time, and the corresponding results are presented in Figure 3. By comparing Figure 3(a-2–c-2), it is evident that an increase in irradiation intensity leads to a significant advancement in the time interval of the rapid precipitation of the α’ phase. Higher irradiation intensity results in an augmented vacancy concentration within the simulated space, thereby enhancing mobility and accelerating Cr aggregation efficiency. However, as the simulation time progresses further, there are no substantial differences observed in the distribution characteristics of the Cr-rich α’ phase under different irradiation intensities. This can be attributed to the sufficient precipitation of Cr. Hence, it can be inferred that increased irradiation intensity primarily accelerates the precipitation rate without significantly altering precipitation morphology [41]. The impact of irradiation on precipitation behavior can thus be ascribed to its influence on the rate of the Ostwald ripening process [42].

**Figure 4:**
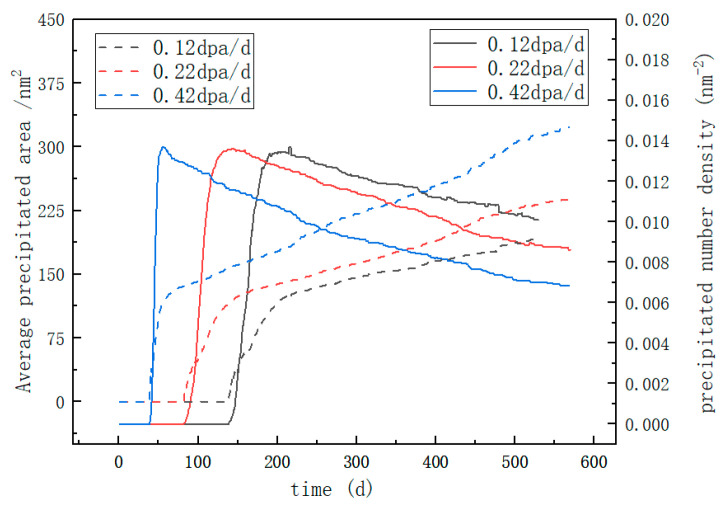
Average area and number density of the α’ precipitates with an average Cr concentration (the dotted line and the left axis indicate the average area of the precipitated phase, and the realization and the right axis indicate the amount of the precipitated phase).

To quantitatively analyze the distribution characteristics of Cr-rich α’ precipitates under different irradiation intensities, the precipitation distribution characteristics of Fe-15Cr-6Al in single crystals at varying levels of irradiation intensity are plotted as illustrated in Figure 4. The simulation results demonstrate the Ostwald curing rate of the precipitates. For convenience, the spot with the maximum number of precipitates in Figure 4 is named the initial peak of the system. As the average Al concentration increased, the amplitude of the initial peak increased with Fe-15Cr-6Al, as shown in Figure 4. In addition, after the initial peak, the number of the α’ precipitates decreased significantly in the high Al concentration group in comparison to that of the low Al concentration group. According to Figure 4, the precipitation time of the precipitated phase under the irradiation intensity of 0.42 dpa/d falls within the range of 40–55 days. Under the irradiation intensity of 0.22 dpa/d, the primary precipitation time for the precipitated phase is observed to be between 89 and 122 days. At the irradiation intensity of 0.12 dpa/d, the main precipitation time for the precipitated phase extends from 148 to 196 days. It can be observed that higher irradiation intensities not only advance the onset of precipitation for the precipitated phase but also significantly reduce the duration of alloy matrix precipitation [43]. The irradiation rate, similar to temperature, is a contributing factor in accelerating the Ostwald ripening process of the Cr-rich phase in Fe-Cr-Al alloys [42]. It is evident that an increase in the irradiation rate will undoubtedly expedite the degradation of the mechanical properties of Fe-Cr-Al.

**Figure 5:**
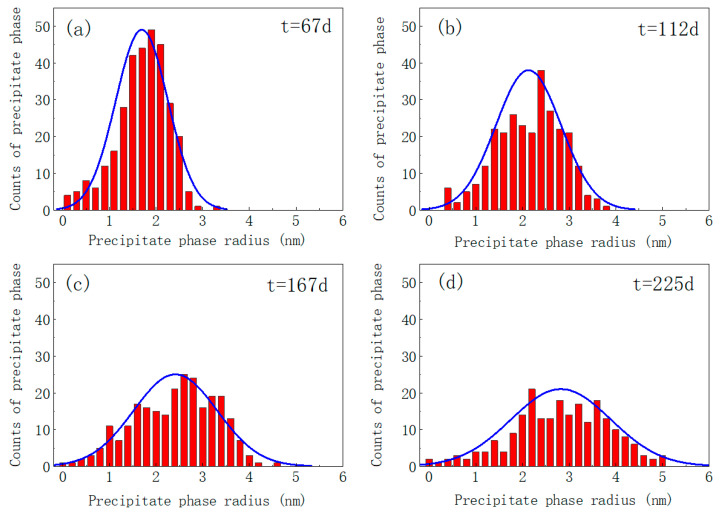
Histograms of radius distribution of Cr-riche α’ precipitates at different times (subfigures (**a**–**d**) represent the distribution after 67 d, 112 d, 167 d, and 225 d, respectively. The red bar chart illustrates the distribution of precipitate phase radius, and the blue curve represents the fitting of normal distribution).

Histograms of the radius distribution of Cr-rich α’ precipitates under the irradiation strength of 0.42 dpa/d at different times are shown in Figure 5. As time increased, the radius distribution of the precipitated phase gradually expanded while the total quantity decreased. At a simulation time of 67 days, the average radius of the precipitated phase was measured to be 1.873 nm with a total count of 313 particles. Upon reaching a simulation time of 225 days, the average radius of the precipitated phase increased to 2.802 nm accompanied by a decrease in the total count to 207 particles. The histogram distribution is in accordance with the fitting curve, suggesting that the distribution of precipitates’ radii approximately follows a normal distribution. It is evident that over time, the proportion of precipitated phases with smaller precipitate radii decreases (Figure 5a accounts for 12%, Figure 5d accounts for 4.7%). This phenomenon can be attributed to the enrichment of the Cr element in the already formed precipitated phases, leading to an increased prevalence of regions with lower Cr content and subsequently hindering the further formation of new precipitates. Experimental evidence also supports this conclusion [43]. According to the radius distribution of the precipitated phase, long-time aging eventually leads to dissolution of the precipitated phase within 2 nm. This observation is further substantiated by pertinent experimental evidence [10].

### 3.2. Effect of Grain Boundary on Segregation

**Figure 6:**
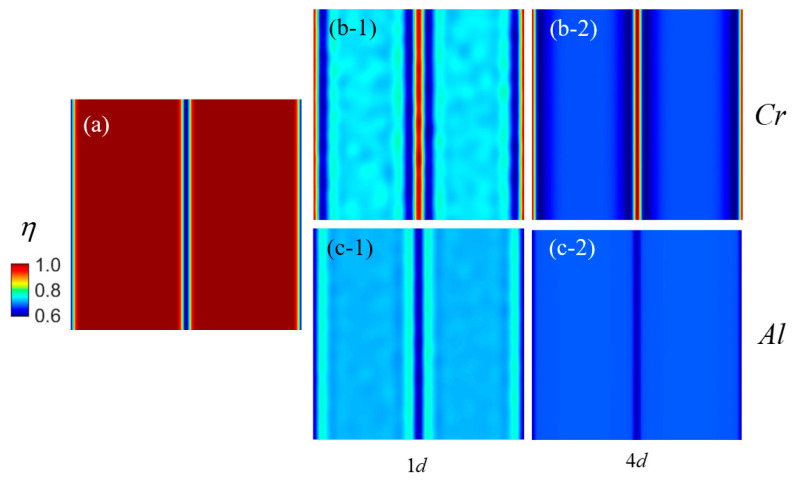
Segregation of Cr and Al in Fe-Cr-Al alloys at (**a**) twin GBs ((**b-1**,**b-2**) represent the distribution of Cr after 1 d and 4 d. (**c-1**,**c-2**) represent the distribution of Al after 1 d and 4 d). Note that the blue region denotes a lower relative concentration of elements, whereas the red areas indicate a higher relative concentration of elements along the GB.

The GB is a crucial factor influencing the localized accumulation of the Cr element in stainless steel [44]. In the case of stainless steel with a chromium concentration below 15%, the occurrence of in-grain precipitation has been found to be challenging, while segregation along the grain boundary remains the primary phenomenon. In this part, a phase-field model is established to simulate the precipitation of Cr-rich precipitates along GBs in Fe-10Cr-6Al by incorporating GB term free energy. Figure 6 presents the distribution results of segregation morphology for twin crystals and Fe-10Cr-Al near their GBs at different time points. In Figure 6, in Patki et al., [16], the C06M steel which has the same composition as Fe-10Cr-6Al is tested after being aged for 1 h with an irradiation strength of 9.3 × 10^−7^ dpa/s. The same experimental conditions were imported into this simulation. Details on the precipitation of Fe-10Cr-6Al stainless steel are attached to Figure 6a. The spatial distribution of Cr and Al elements simulated by this model after different time are shown in (b-1), (b-2) and (c-1), (c-2) in Figure 6, respectively. The simulation results of this model are similar to the experimental results. It is evident that Cr segregation on the GB is significantly more pronounced than within the grains, rendering the precipitated phase less discernible within the same contrast group. On the Cr-rich GBs, a simultaneous increase in Cr concentration and decrease in Al concentration are observed, consistent with the composition content of Cr-rich precipitated phases within grains. On a time scale of less than 10 days, the presence of GBs leads to the formation of chromium-enriched regions and aluminum-depleted regions along the GBs, as well as chromium-depleted regions and aluminum within approximately 5 nm proximity to the GBs [16].

**Figure 7:**
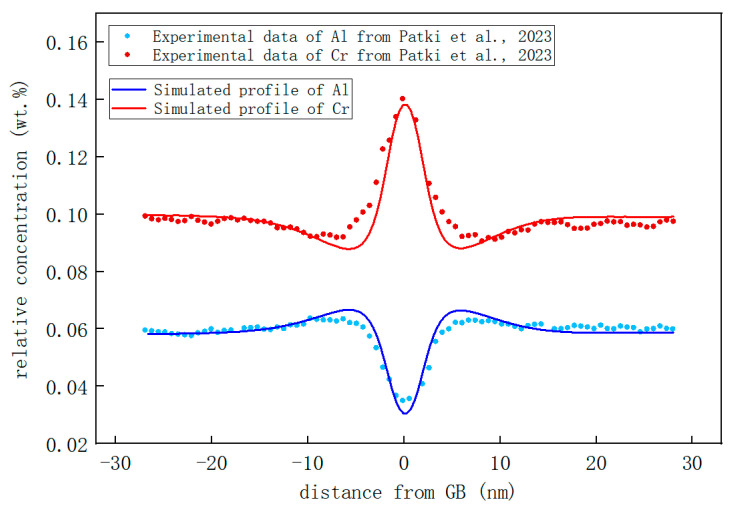
Concentration profile of segregation of Cr and Al in Fe-Cr-Al alloys at twin GBs and comparison with experimental results from Patki et al., 2023 [16].

The composition distribution curves of Cr and Al near the GBs were further drawn and compared with experimental work [16], as illustrated in Figure 7. It is evident that the segregation of Cr and Al near the GB is well replicated by the model established in this study, and the concentration distribution closely resembles that observed experimentally. In terms of GB segregation for Fe-10Cr-6Al, there is an increase in Cr at the GB with a maximum concentration reaching ~13.54%, while Al concentrations decreased to a minimum value of ~3.38%. The simulation results show that under the same conditions, the maximum Cr segregation concentration at the GB is 3.542% higher than the original Cr concentration in the alloy matrix. This difference very close to the 3.7% observed by [16]. The concentration of Al at the GB exhibits a 2.2% reduction compared to that in the alloy matrix. This result slightly surpasses the 1.8% discrepancy reported by the experiment, adequately demonstrating the robustness of this model’s calculations. The simulation results also revealed the presence of Cr depletion zones and Al enrichment zones adjacent to GBs, which exhibited a remarkable agreement with the experimental findings. Moreover, the simulation results demonstrate a reduction in the concentration of Cr within the depleted zone by approximately 0.92% compared to that in the alloy matrix, while revealing an increase in the concentration of Al within the enriched zone by about 0.83% relative to that in the alloy matrix. This result is also in good agreement with the results obtained in experiment [16].

### 3.3. Element Segregation in Multigrain Fe-Cr-Al

**Figure 8:**
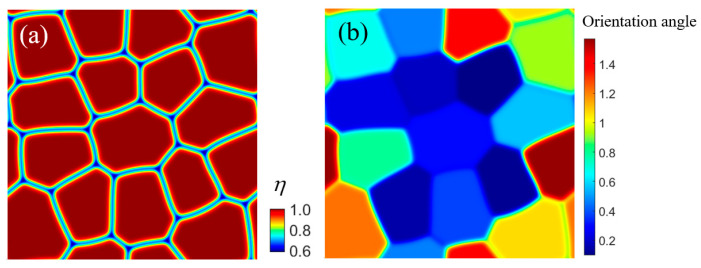
The initial polycrystalline structure of Fe-Cr-Al ((**a**) for GB distribution, (**b**) for grain orientation distribution).

The model established in this study is employed for precipitates of Fe-15Cr-6Al alloy in a polycrystalline system, aiming to analyze its precipitation under long-term irradiation with an irradiation intensity of 0.12 dpa/d. To obtain a polycrystalline structure suitable for phase-field simulation, we calculated a polycrystalline system based on the polycrystalline grown phase-field model. The morphology and orientation of the polycrystals, as well as their GBs, are illustrated in Figure 8. The polycrystal system comprises 16 grains exhibiting diverse orientations, with the angle distribution of the grain orientation ranging from 0 to π/2. Periodic boundary conditions were implemented for each boundary, and the simulation size encompassed a grid of 512 × 512.

**Figure 9:**
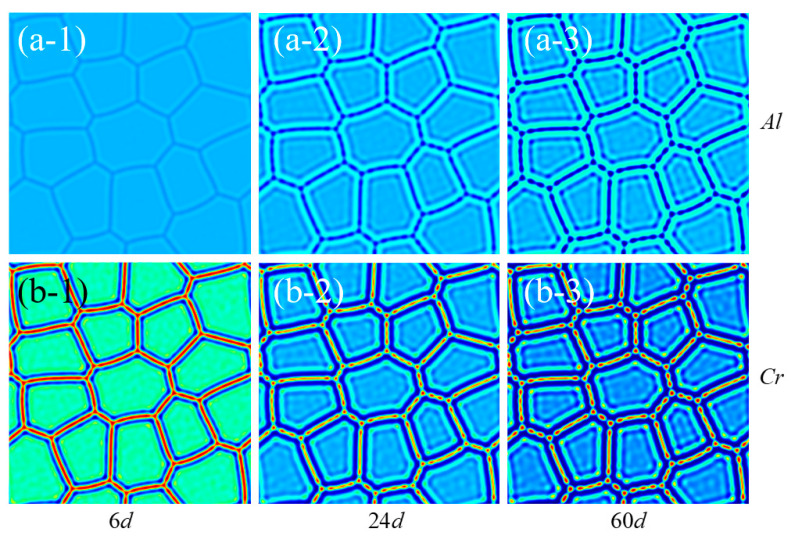
Concentration profile of segregation of Cr and Al in Fe-Cr-Al alloys in a multi-grain system (**a-1**–**a-3**,**b-1**–**b-3**) represent the concentration distribution of Cr and Al after different simulation times. The blue region denotes a lower concentration of elements, whereas the red areas indicate a higher concentration of elements along the GB.

The simulation results are presented in Figure 9. According to the simulation results, it is evident that Cr-rich precipitates are formed along the GBs, particularly on the three-bifurcated GBs, after precipitation for 60 days. The precipitated phase exhibits a spindle or oval shape with slight elongation along the GB. Concurrently, there is an obvious decrease in Al concentration at the same location. The comparison of the results presented in Figure 9 and Figure 2 proves that the presence of GBs leads to a reduction in the quantity of precipitated phase [41]. In addition, the presence of GBs leads to a decrease in both the amount of the precipitated phase within the grains and the concentration of Cr in the core of these precipitates. This phenomenon can be attributed to the segregation of Cr along the GBs [17]. Comparing the results of Figure 3, segregation with GBs takes less time than precipitation in single crystals. Under identical temperature and irradiation conditions, the former requires approximately 150 days for the formation of the precipitated phase, whereas the latter accomplishes the development of a high-concentration Cr-rich region on the grain boundary in just about 20 days. Overall, GBs have a dual effect on the precipitate of Fe-Cr-Al alloy. On one hand, GBs reduce the quantity of the precipitated phase within individual grains; on the other hand, GBs promote faster segregation along GBs. The presence of grain boundaries has two effects on the mechanical properties of Fe-Cr-Al alloy. It reduces the amount and size of precipitated phases within the crystal, thereby potentially mitigating brittle fracture under irradiation. However, the existence of Cr-rich precipitates at grain boundaries may impact their slip ability, leading to a reduction in the overall plasticity of the alloy.

## 4. Conclusions

The present work establishes an amplitude-modulated phase-field model of Fe-Cr-Al alloy, incorporating irradiation and a polycrystalline structure. The main conclusions are as follows:

(1) The phase-field model enables the simulation of the acceleration effect of irradiation on precipitation and segregation, while elucidating the role of GBs in segregation. By introducing an irradiation vacancy source term related to irradiation intensity, the model enables the simulation of the irradiation-accelerated precipitation and segregation. Moreover, the model integrates polycrystalline grain boundaries to effectively depict the segregation of Fe-Cr-Al alloy along GBs under irradiation conditions.
(2) The precipitation of the Cr-rich α’ second phase in Fe-Cr-Al alloy is simulated under varying irradiation intensities. The time and radius distributions of Cr-rich precipitates under different irradiation intensities were calculated. The results show that after 225 days at the irradiation intensity of 0.42 dpa/d, the average precipitation radius of the Fe-15Cr-6Al alloy is 2.802 nm and the number density is ~0.0147 nm^−2^. Long-time aging eventually leads to the dissolution of the precipitated phase within 2 nm.
(3) The segregation of Cr and Al in Fe-Cr-Al is simulated in both twin and polycrystalline systems. Segregation at GBs leads to the formation of Cr-rich and Al-poor regions with a width of ~14 nm. Under the influence of an irradiation intensity of 0.12 dpa/d, the precipitation of Cr mainly happening in ~150 days is replaced with segregation mainly happening in ~20 days. The simulation results demonstrate excellent agreement with the corresponding experimental findings.

## Figures and Tables

**Table 1:** List of Gibbs free energy parameters of Fe-Cr-Al alloys.

Parameter	Value	Reference
*G_Fe_* ^0^	1225.71 + 124.09*T* − 23.49*T*In*T* − 4.40e^−3^*T*2 − 5.88e^−8^*T*^3^ + 77,358T^−1^ J/m^3^	[31]
*G_Cr_* ^0^	−8856.90 + 157.50*T* − 26.90*T*In*T +* 1.89e^−3^*T*2 − 1.48e^−6^*T*^3^ + 139,250*T*^−1^ J/m^3^	[31]
*G_Al_* ^0^	−1193.24 + 218.24T − 38.58*T*In*T +* 1.85e^−2^*T*2 − 5.64e^−6^*T*^3^ + 741*T*^−1^ J/m^3^	[31]
*L_FeCr_*	20,500 − 9.68*T*	[32]
*L_FeAl_*	−54,900 + 10.00*T*	[32]
*L_CrAl_*	−122,452.90 + 31.65*T*	[32]

**Table 2:** List of diffusion coefficients of Fe-Cr-Al alloys.

Parameter	Value	Reference
*D_Fe_*	$2.8\times 10^{-4}\exp\left(-251\left(kJ/mol\right)/RT\right)$	[32]
*D_Cr_*	$3.7\times 10^{-3}\exp(-267\left(kJ/mol\right)/RT)$	[32,37]
*D_Al_*	$5.2\times 10^{-4}\exp(-246\left(kJ/mol\right)/RT)$	[32]
*D_v_*	$3.84\times 10^{-4}\exp(-300\left(kJ/mol\right)/RT)$	[28,38]
*D_i_*	$2.05\times 10^{-4}\exp(-280\left(kJ/mol\right)/RT)$	[28,38]

## Data Availability

The data that support the findings of this study are available from the corresponding authors upon request.
